# BiGvCL: bipartite graph-based cross-domain contrastive learning model for the predicting drug-gene interactions

**DOI:** 10.1093/bib/bbaf710

**Published:** 2026-01-28

**Authors:** Shida He, Zixu Wang, Jing Li, Quan Zou, Feng Zhang

**Affiliations:** The Joint Innovation Center for Engineering in Medicine, Quzhou Affiliated Hospital of Wenzhou Medical University, Quzhou People's Hospital, No. 100, Minjiang Avenue, Kecheng District, Quzhou, Zhejiang, 324000, China; Department of Respiratory and Critical Care, Quzhou Affiliated Hospital of Wenzhou Medical University, No. 100, Minjiang Avenue, Kecheng District, Quzhou, Zhejiang, 324000, China; Yangtze Delta Region Institute (Quzhou), University of Electronic Science and Technology of China, No. 1 Chengdian Road, Kecheng District, Quzhou, Zhejiang, 324000, China; College of Computer Science and Electronic Engineering, Hunan University, Lushan South Road, Yuelu District, Changsha, Hunan, 410082, China; Department of Microbiology, University of Hong Kong, Block T, Queen Mary Hospital, Pok Fu Lam Road, Pok Fu Lam, Hong Kong, 000000, China; Yangtze Delta Region Institute (Quzhou), University of Electronic Science and Technology of China, No. 1 Chengdian Road, Kecheng District, Quzhou, Zhejiang, 324000, China; The Joint Innovation Center for Engineering in Medicine, Quzhou Affiliated Hospital of Wenzhou Medical University, Quzhou People's Hospital, No. 100, Minjiang Avenue, Kecheng District, Quzhou, Zhejiang, 324000, China; Department of Respiratory and Critical Care, Quzhou Affiliated Hospital of Wenzhou Medical University, No. 100, Minjiang Avenue, Kecheng District, Quzhou, Zhejiang, 324000, China

**Keywords:** drug-gene interactions, graph neural networks, contrastive learning, cross-domain knowledge transfer, transductive learning

## Abstract

Drug-gene interactions (DGIs) influence the toxicity or ineffectiveness of the drug therapy and play an important role in elucidating drug mechanisms, predicting potential adverse effects, and facilitating precision medicine. Existing computational methods typically rely on chemical or genetic sequence features of drugs and genes, limiting their effectiveness for novel entities lacking explicit annotations. To address this, we propose BiGvCL, a framework that predicts DGIs exclusively based on network topology, requiring no explicit feature information for drugs or genes. BiGvCL introduces a lightweight graph attention mechanism (GATLite) to efficiently aggregate local neighborhood information. Additionally, we develop a gated graph convolutional network (GatedGCN) to explicitly learn high-order interactions between drugs and genes, further integrating contrastive learning to enhance the model’s generalizability. Comprehensive experiments on DrugBank and DGIdb datasets show that BiGvCL achieves competitive performance across all metrics compared with representative baselines. Cross-domain evaluations on OGB datasets further confirm its adaptability to heterogeneous biomedical networks. Ablation and hyperparameter analyses highlight the key contributions of contrastive and gated mechanisms, while case studies and molecular docking provide supporting evidence for the biological relevance of predictions. Collectively, while BiGvCL is constrained by its reliance on network topology and transductive learning paradigm, it demonstrates the potential of topology-based approaches for discovering novel drug-gene interactions, which may inform drug repurposing and precision medicine efforts.

## Introduction

Bioinformatics plays a pivotal role in interpreting vast amounts of data generated from transcriptomics, genomics, proteomics, and other omics fields, facilitating the understanding of biological processes and predicting gene functions [1–4]. Numerous bioinformatics tools have successfully elucidated disease mechanisms underlying infectious diseases and various cancers, including colon, gastric, bladder, prostate, and lung cancers [4–8]. Some computational drug-target interaction (DTI) prediction was established through early works on supervised chemogenomic inference [9], bipartite local models [10], and statistics approaches [11], providing the methodological basis for subsequent advances. In recent years, these bioinformatics methodologies have increasingly integrated artificial intelligence (AI), particularly deep learning (DL), contributing to advances in drug discovery by addressing significant time, cost, and risk traditionally associated with drug development [12–21]. Techniques such as deep generative models, notably diffusion models, facilitate efficient exploration of chemical spaces, enabling rapid design of drug candidates with desired pharmacological profiles, thus enhancing diversity and efficacy in drug design [22–30]. In protein structure prediction, AI-driven models like AlphaFold [31] have resolved long-standing challenges in structural biology, accelerating target structure elucidation and structure-based drug design [32–34]. In biological sequence modeling, deep neural networks also show potential, e.g. Zhao *et al.* [35] conducted sequence-based toxicity prediction using CNN + GRU with channel attention and a variational information bottleneck, and Le *et al.* [36] presented sequence-based identification of vesicular transport proteins using GRU with PSSM profiles and class-weighting for imbalance. Moreover, DTI prediction has advanced significantly, with graph neural networks, including GraphDTA and GraphormerDTI, effectively integrating molecular structures and protein sequences to achieve competitive performance on benchmark datasets [37–41]. In particular, Aragh *et al.* [42] proposed MiRAGE-DTI, which integrates multiple similarity measures of drugs and targets and employs a Random Forest classifier to predict drug–target interactions. Gao *et al.* [43] developed HMT-DTI, a precomputed hierarchical meta-path learning framework that adopts a Transformer-based message-passing mechanism to assess the importance of neighboring nodes and adaptively aggregate meta-path information. Through a hierarchical knowledge extraction strategy, HMT-DTI evaluates the significance of multi-hop neighbors and diverse meta-path patterns, thereby capturing rich semantic representations of drugs and targets.

Despite progress in DTI models, they predominantly focus on direct binding relationships between drugs and protein targets, neglecting broader systemic effects within cellular gene regulatory networks. In contrast, drug-gene interactions (DGIs) offer a more comprehensive perspective by capturing drug impacts on gene expression, transcriptional regulation, and epigenetic modifications, thereby illuminating multi-target mechanisms, adverse effect formation, and individual variability in patient responses [44–50]. However, the inherent complexity and diversity of DGI networks, characterized by various interaction types (e.g. agonism, antagonism, upregulation, downregulation), render exhaustive experimental validation prohibitively expensive and time-consuming.

With advancements in graph representation learning, researchers increasingly leverage graph structure to predict DGIs [51–53]. Notably, the CoSMIG model employs communicative subgraph inference for inductive predictions in multi-relational drug-gene networks [54, 55], while MDTips [39] integrates multimodal data such as knowledge graphs, gene expression profiles, and molecular structures, demonstrating robustness across diverse data scenarios. Furthermore, HGDruG constructs multi-task prediction frameworks based on heterogeneous hypergraphs capturing micro-to-macro scale drug attributes [56]. Graph-based approaches have also excelled in drug repositioning, exemplified by the graph foundation model TxGNN, which improves accuracy in zero-shot drug repurposing across numerous disease contexts [57].

Despite such advancements, current AI-driven DGI prediction models face critical challenges [58–60]: (i) pronounced data sparsity limits generalization due to scant known interactions; (ii) models often address narrowly defined problems without comprehensive capabilities; and (iii) integrating heterogeneous and multimodal data introduces complexity, imbalanced data distributions, and long-tail phenomena, complicating precise predictions. Recent approaches, including dynamic hypergraph-based DGCL [61] and singular-value decomposition-enhanced SGCLDGA [62] have partly mitigated these issues but remain limited in scalability, generalizability, and predictive specificity. In addition, there are other challenges in the study of drug–gene interactions. For example, the graph diffusion network (GDNDGP) proposed by Wu *et al.* [63] aims to predict whether an association exists between drugs and genes in a heterogeneous biomedical graph; however, the model only outputs the probability of an interaction and cannot distinguish specific interaction types. He *et al.* [64] proposed a novel inductive learning-based model to predict unseen drug–gene interactions by constructing a multi-relational drug–disease–gene (DDG) graph. However, the model heavily relies on domain-specific knowledge and, similarly, only predicts the existence of interactions without identifying their specific types or underlying mechanisms.

To address these limitations, we propose BiGvCL, a bipartite graph-based cross-domain contrastive learning framework relying solely on network topology. Our approach captures both local and global interaction information without explicit feature annotations. The primary contributions of this study include:

1. Introducing lightweight graph attention network (GATLite), a lightweight attention mechanism for adaptive neighborhood aggregation, maintaining inductive learning capabilities and computational efficiency while reducing parameter complexity.
2. Developing the gated graph convolutional network (GatedGCN) module, which explicitly modeling interaction features via associative matrix-based propagation, adaptively integrating inherent node features with structural information, well-suited to bipartite networks and complex nonlinear relationship modeling.
3. Designing a contrastive learning framework to guide dual-graph structure extraction and enhance the discriminative capacity of drug and gene representations.
4. Conducting comprehensive experiments on benchmark datasets (DrugBank [65], DGIdb [66], LINCS L1000 [67], OGB [68] drug-disease), including ablation and sparsity analyses, validating BiGvCL’s potential in DGI prediction tasks.
5. Demonstrating BiGvCL’s practical utility through case studies confirming its capability to identify literature-supported novel drug-gene interactions, highlighting its role in drug discovery and repositioning.

## Materials and methods

### Datasets

This study employed four representative datasets covering drug-gene and drug-disease interactions to evaluate the model’s predictive performance and generalizability across multiple scenarios. The detail are provided in Table 1, the DrugBank dataset containing drug-gene interactions involving 425 drugs and 11 284 genes, comprising a total of 80 924 interactions categorized into upregulation and downregulation. The DGIdb dataset includes 1185 drugs and 1664 genes, totaling 11,366 interactions spanning 14 complex relationship types (e.g. binder, inhibitor, agonist), which can assess the model’s capability to distinguish multiple pharmacological relationships. The LINCS L1000 dataset consists of 1878 drugs and 3769 genes, with a total of 29 610 interaction pairs, categorized as upregulation or downregulation of gene expression, serving as an independent external dataset to further evaluate the model’s cross-dataset generalization. Additionally, to assess the model’s performance in a cross-domain heterogeneous graph environment, we selected the OGB biokg knowledge graph dataset, which includes 686 drugs, 1507 diseases, and 10 294 drug-disease interactions simply categorized as ‘interaction’ or ‘no interaction.’

**Table 1:** Summary of datasets used in this study.

Dataset	Drugs	Genes/Diseases	Interactions	Classes
DrugBank	425	11,284(G)	80 924	2
DGIdb	1185	1664(G)	11 366	14
LINCS L1000	1878	3769(G)	29 610	2
OGB biokg	686	1507(D)	10 294	2

Note: DrugBank: Two interaction categories (up-regulation and down-regulation).

DGIdb: Fourteen interaction categories including binder, inhibitor, agonist, partial agonist, antibody, antagonist, potentiator, cofactor, positive modulator, activator, modulator, allosteric modulator, blocker, and ligand.

LINCS 1000: Two interaction categories (up-regulation and down-regulation).

OGB biokg: Two interaction categories (interaction, no interaction).

### Data preprocessing and graph construction

For fair comparison, we adopted the drug-gene data splits provided by DGCL [61], maintaining an ~4:1 train-test ratio. Given sufficient data volume, we randomly sampled 10% of the training set as a validation set. All experiments were repeated across five independent runs with different results to ensure statistical robustness. Notably, the LINCS L1000 dataset served exclusively as an external validation set to assess cross-dataset generalizability. Negative samples in the OGB dataset were generated using a frequency-smoothed sampling strategy. This approach preferentially selected low-frequency nodes while suppressing high-frequency ones, independently sampling node pairs from weighted distributions. Generated drug-disease pairs that did not overlap with positive samples and contained no duplicates were labeled as negative samples, thus addressing class imbalance and data sparsity inherent to interaction prediction tasks to a certain extent.

Due to the transductive learning paradigm, drug and gene nodes in the validation and test sets must appear in the training graph; however, no interaction edges (drug-gene pairs) overlap between training and evaluation splits, thereby preventing data leakage while enabling the model to leverage shared node representations. For baseline methods requiring explicit feature inputs (e.g. GRALS [69]), we collected molecular structural information for drugs from DrugBank and PubChem [70] databases and retrieved gene expression profiles from the GTEx [71] database. For drugs or genes lacking publicly available feature data, we employed randomly initialized embedding vectors as substitutes to ensure compatibility across all baseline comparisons.

### Model architecture

**Figure 1:**
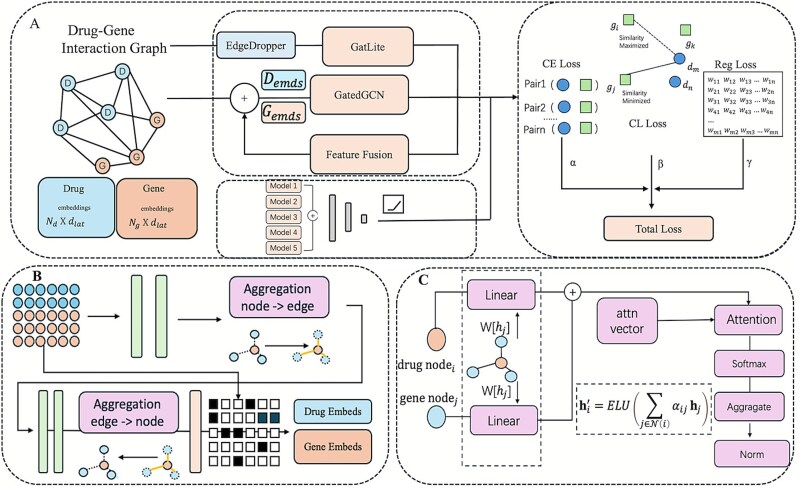
Overview of BiGvCL architecture. (A) Framework outline with embeddings and loss functions. (B) GatedGCN mechanism. (C) GATLite attention module.

To address challenges associated with DGI prediction, we propose the BiGvCL framework, as depicted in Fig. 1. This framework comprises three core components: (i) a GATLite designed to capture structural graph information; (ii) a GatedGCN that explicitly learns higher-order interaction patterns; (iii) a contrastive learning strategy to enhance the discriminative capability of learned representations. Furthermore, we integrate multiple model variants through an ensemble learning approach to improve prediction performance and stability.

#### Problem definition

We define the prediction of DGIs as a multi-label classification task. Formally, given a set of drugs ${V}_d=\left\{{d}_1,{d}_2,\dots, {d}_m\right\}$, a set of genes ${V}_g=\left\{{g}_1,{g}_2,\dots, {g}_n\right\}$, and known interactions $E\subseteq{V}_d\times{V}_g\times R$, where $R$ denotes interaction types, our aim is to learn a predictive function to infer interaction types for unknown drug-gene pairs:


$$ f:{V}_d\times{V}_g $$

To facilitate efficient graph-based modeling, we first unify drug and gene entities into a single node set and re-index them through a bijection to consecutive integers. Subsequently, an adjacency matrix ${A}_{\mathrm{full}}$representing the bipartite interactions is constructed:


$$ {A}_{\mathrm{full}}=\left[\begin{array}{@{}cc@{}}{0}_{\mid D\mid \times \mid D\mid }& R\\{}{R}^T& {0}_{\mid G\mid \times \mid G\mid}\end{array}\right],\ {R}_{ij}=\left\{\begin{array}{@{}c@{\ \ }c@{}}1,&\ \mathrm{if}\ \mathrm{interaction}\ \left({d}_i,{g}_j\right)\ \mathrm{exists}\ \\{}0,&\ \mathrm{otherwise}\ \end{array}\right. $$

To capture node-specific information and ensure stable propagation during graph convolution operations, we introduce self-loop connections, yielding an augmented adjacency matrix:


$$ \overset{\sim }{A}={A}_{\mathrm{full}}+{I}_N $$

Finally, we apply symmetric normalization to mitigate degree imbalances among nodes:


$$ \hat{A}={D}^{-\frac{1}{2}}\overset{\sim }{A}{D}^{-\frac{1}{2}} $$

Here, $D$ is a diagonal degree matrix derived from $\overset{\sim }{A}$. This normalized adjacency matrix is then converted into a sparse tensor as input to graph neural networks.

#### Lightweight graph attention network

Graph attention networks [72, 73] (GATs) exhibit have been widely used for node representation but encounter limitations in computational efficiency and memory usage when applied to large-scale biological molecular networks. To address these issues, we introduce a lightweight variant termed GATLite. The core innovation of GATLite lies in decoupling feature transformation from attention computation through a shared linear projection matrix. Specifically, node features are first transformed through a linear mapping $W\in{R}^{F^{\prime}\times F}$ to generate intermediate node representations:


$$ {\mathbf{z}}_i=W{\mathbf{h}}_i $$

Next, for a node $i$ with neighbors $\mathcal{N}(i)$, we compute and normalize the attention coefficients:


$$ {e}_{ij}= LeakyReLU\left({\mathbf{a}}^T\left[{\mathbf{z}}_i\parallel{\mathbf{z}}_j\right]\right) $$

$$ {\alpha}_{ij}=\frac{\exp \left({e}_{ij}\right)}{\sum_{k\in \mathcal{N}(i)}\exp \left({e}_{ik}\right)} $$

Here, $\mathbf{a}\in{\mathrm{R}}^{2{F}^{\prime }}$ is the attention parameter vector, and $\parallel$ represents feature concatenation.

Finally, node representations are aggregated using the computed attention weights, followed by applying an ELU activation function and L2 normalization to enhance representation capability and stabilize training:


$$ {\mathbf{h}}_i^{\prime }=L2 Norm\left( ELU\left(\sum_{j\in \mathcal{N}(i)}{\alpha}_{ij}{\mathbf{z}}_j\right)\right) $$

The implementation leverages sparse matrix operations to ensure computational efficiency on large-scale networks, with attention dropout applied during training to prevent overfitting. This design maintains the expressive power of attention mechanisms while reducing computational overhead.

#### Gated graph convolutional network

Drug-gene interaction networks typically involve complex, higher-order relational patterns that are difficult to capture through simple neighborhood aggregation. To address this challenge, we designed the GatedGCN module to explicitly model multi-node, second-order dependencies within networks. The core idea of GatedGCN is to integrate gated mechanisms with graph message-passing processes, thus enhancing representation learning capabilities.

The GatedGCN module employs a multi-stage transformation process. First, node features undergo nonlinear transformations through multi-layer perceptrons (MLPs). Bidirectional information propagation is then performed: features flow from nodes to hyperedges, are aggregated and transformed, and subsequently propagate back to nodes. This design enables the capture of higher-order connectivity patterns that extend beyond direct node-to-node relationships.

The gating mechanism adaptively balances node-intrinsic features and structural information through a learnable fusion strategy:


$$ {\mathbf{g}}_i=\sigma \left({W}_g\left[{\mathbf{h}}_i\parallel{\mathbf{z}}_i^{\prime}\right]+{\mathbf{b}}_g\right) $$

$$ {\mathbf{h}}_i={\mathbf{g}}_i\odot{\mathbf{z}}_i+\left(1-{\mathbf{g}}_i\right)\odot{\mathbf{h}}_i $$

where ${\mathbf{h}}_i\in{\mathbb{R}}^d$ denotes the original feature vector of node $i,{\mathbf{z}}_i^{\prime}\in{\mathbb{R}}^d$ represents the aggregated higher-order information, ${W}_g\in{\mathbb{R}}^{d\times 2d}$ is the learnable weight matrix for the gating mechanism, ${\mathbf{b}}_g\in{\mathbb{R}}^d$ is the bias term, $\left[\cdotp \parallel \cdotp \right]$ denotes concatenation, $\odot$ represents element-wise multiplication, and $\sigma$ denotes the sigmoid activation function. This adaptive fusion allows the model to dynamically determine the contribution of structural patterns versus intrinsic node features for each entity.

The GatedGCN module complements GATLite, with the former capturing global higher-order relational patterns and the latter focusing on local neighborhood structures. The integration of both strategies enhances the model’s capacity to represent complex drug-gene interaction patterns.

#### Contrastive learning framework

Contrastive learning has emerged as a paradigm in representation learning research, with the normalized temperature-scaled cross-entropy (NT-Xent) loss attracting particular attention due to its application in frameworks such as SimCLR [74]. However, the standard NT-Xent loss assumes a single positive sample for each anchor, restricting its potential applicability in multi-relational domains. In this study, we propose the Multi-Positive NT-Xent loss, an extension of the standard NT-Xent loss designed to effectively handle scenarios where multiple samples can be considered as positive pairs. The standard NT-Xent loss is formally defined as:


$$ {\mathcal{L}}_{\mathrm{InfoNCE}}=-\frac{1}{\mid N\mid}\sum_{i=1}^{\mid N\mid}\log \frac{\exp \left( sim\left(i,{p}_i\right)/\tau \right)}{\exp \left( sim\left(i,{p}_i\right)/\tau \right)+\sum_{j\ne{p}_i}\exp \left( sim\left(i,j\right)/\tau \right)} $$

where ${p}_i$ is the single positive sample corresponding to anchor $i$, and $\tau$ denotes the temperature parameter.

The proposed Multi-Positive NT-Xent loss generalizes this formulation as follows:


$$ {\mathcal{L}}_{\mathrm{Multi}-\mathrm{Positive}}=-\frac{1}{\mid N\mid}\sum_{i=1}^{\mid N\mid}\log \frac{\sum_{j\in P(i)}\exp \left( sim\left(i,j\right)/\tau \right)}{\sum_k\exp \left( sim\left(i,k\right)/\tau \right)} $$

where $P(i)$ denotes the set of samples that form positive pairs with anchor $i$.

The Multi-Positive NT-Xent loss leverages the inherent structure in biological networks by recognizing that entities may have multiple valid representations. Through an adaptive masking mechanism, the framework identifies and aggregates contributions from all relevant positive associations. This approach enables the model to learn representations that capture the multifaceted nature of drug-gene interactions, where the same biological entity may manifest through different molecular profiles or experimental conditions. The temperature-scaled similarity computation ensures that the model maintains discriminative power while accommodating the natural variability within entity groups.

#### Loss function

In training the BiGvCL model, we employed a composite loss function designed to enhance the model’s representational robustness and generalization. Specifically, the combined loss function integrates three components: regularization loss, contrastive learning loss, and cross-entropy loss.

The regularization loss helps prevent model overfitting by encouraging smaller weight values, defined as:


$$ {\mathcal{L}}_{\mathrm{reg}}=\sum_{W\in \theta}\parallel W{\parallel}_2^2 $$

where $\theta$ represents the set of all model parameters, and $\parallel W{\parallel}_2^2$ denotes the squared L2 norm of parameter $W$.

The contrastive learning loss, implemented via MultiPosNCE, enhances the discriminative power of node representations by distinguishing between positive and negative samples:


$$ {\mathcal{L}}_{cl}=-\frac{1}{N}\sum_{i=1}^N\log \frac{\sum_{j\in P(i)}\exp \left( sim\left({\mathrm{h}}_i,{\mathrm{h}}_j\right)/\tau \right)}{\sum_k\exp \left( sim\left({\mathrm{h}}_i,{\mathrm{h}}_k\right)/\tau \right)} $$

The cross-entropy loss ensures accurate prediction of drug-gene interaction labels:


$$ {\mathcal{L}}_{ce}=-\frac{1}{N}\sum_{i=1}^N\sum_{c=1}^C{y}_{i,c}\log \left({\hat{y}}_{i,c}\right) $$

where ${y}_{i,c}$ is the ground-truth label, ${\hat{y}}_{i,c}$ is the predicted probability of node $i$ belonging to class $c$, and $C$ is the number of classes.

The total loss function integrates these individual components using corresponding weighting coefficients $\alpha, \beta$, and $\gamma$:


$$ {\mathcal{L}}_{\mathrm{total}}=\alpha{\mathcal{L}}_{\mathrm{reg}}+\beta{\mathcal{L}}_{cl}+\gamma{\mathcal{L}}_{ce} $$

For optimization, we used the AdamW [75] optimizer paired with a cyclic learning rate [76] scheduler (CyclicLR), initialized at a learning rate of 0.001. This strategic combination effectively balances model convergence speed and training stability, contributing to the performance of BiGvCL in predicting drug-gene interactions.

#### Evaluation metrics

To evaluate the predictive effectiveness of the BiGvCL model, we utilized five standard performance metrics commonly employed in multi-label classification tasks: Accuracy (ACC), Macro-F1 score (Macro-F1), Area Under the Receiver Operating Characteristic Curve (AUROC), Area Under the Precision-Recall Curve (AUPR), and Matthews Correlation Coefficient (MCC).

Accuracy (ACC) quantifies the ratio of correctly predicted instances to the total number of instances, calculated as:


$$ ACC=\frac{TP+ TN}{TP+ FP+ TN+ FN} $$

Macro-F1 computes the mean F1 score across all classes, thereby providing an unbiased metric for datasets with class imbalance. The F1 score for class $i$ is given by:


$$ F{1}_i=\frac{2\times{\mathrm{Precision}}_i\times{\mathrm{Recall}}_i}{\ {\mathrm{Precision}}_i+{\mathrm{Recall}}_i} $$

where ${Precision}_i=\frac{T{P}_i}{T{P}_i+F{P}_i}$ and ${Recall}_i=\frac{T{P}_i}{T{P}_i+F{N}_i}$.

AUROC measures the model’s capability to distinguish between classes across all classification thresholds, specifically assessing the trade-off between true positive rate (TPR, sensitivity) and false positive rate (FPR, 1-specificity). AUPR evaluates the model’s performance by measuring the area under the precision-recall curve, emphasizing predictions for minority (positive) classes, and thus is particularly suited for imbalanced datasets.

Matthews Correlation Coefficient (MCC) provides an comprehensive evaluation metric that considers all aspects of the confusion matrix, defined as:


$$ MCC=\frac{TP\times TN- FP\times FN}{\sqrt{\left( TP+ FP\right)\left( TP+ FN\right)\left( TN+ FP\right)\left( TN+ FN\right)}} $$

In the above definitions, $TP$(true positives) refers to correctly identified positive instances; $TN$(true negatives) represents correctly identified negative instances; $FP$(false positives) denotes negative instances incorrectly identified as positives; and $FN$(false negatives) indicates positive instances incorrectly classified as negatives. The combined use of these metrics provides a comprehensive assessment of the BiGvCL model’s predictive accuracy and generalization ability.

## Results

### Model performance and comparison with baselines

To validate the effectiveness of the proposed BiGvCL model, extensive performance evaluations were conducted on two benchmark datasets, DrugBank and DGIdb. Results were compared with several existing state-of-the-art methods. Tables 2 and 3 report the performance of each method across multiple metrics on both the validation and test sets after five independent repeated experiments.

**Table 2:** Comparison of different methods on the DrugBank dataset.

**Method**	**Features**	**Split**	**Acc**	**Macro_f1**	**AUROC**	**AUPR**	**MCC**
MC	no	val	0.597 ± 0.008	0.596 ± 0.008	0.629 ± 0.006	0.620 ± 0.006	0.193 ± 0.015
GRALS	yes	val	0.633 ± 0.016	0.632 ± 0.016	0.689 ± 0.018	0.664 ± 0.018	0.268 ± 0.032
F-EAE	no	val	0.591 ± 0.005	0.576 ± 0.008	0.636 ± 0.003	0.631 ± 0.002	0.197 ± 0.007
GC-MC	yes	val	0.648 ± 0.003	0.648 ± 0.003	0.678 ± 0.003	0.650 ± 0.003	0.296 ± 0.005
sRGCNN	yes	val	0.641 ± 0.002	0.640 ± 0.002	0.671 ± 0.002	0.649 ± 0.004	0.281 ± 0.004
PinSage	yes	val	0.648 ± 0.006	0.647 ± 0.006	0.716 ± 0.002	0.719 ± 0.002	0.297 ± 0.009
IGMC	no	val	0.643 ± 0.003	0.636 ± 0.009	0.715 ± 0.001	0.720 ± 0.002	0.297 ± 0.003
CosMIG	no	val	0.670 ± 0.006	0.670 ± 0.006	0.740 ± 0.005	0.742 ± 0.005	0.340 ± 0.011
DGCL	no	val	0.684 ± 0.004	0.684 ± 0.004	0.737 ± 0.003	0.715 ± 0.003	0.368 ± 0.009
**BiGvCL**	no	val	**0.711 ± 0.002**	**0.710 ± 0.002**	**0.772 ± 0.001**	**0.751 ± 0.002**	**0.421 ± 0.004**
MC	no	test	0.623 ± 0.003	0.624 ± 0.003	0.685 ± 0.006	0.695 ± 0.002	0.247 ± 0.006
GRALS	yes	test	0.631 ± 0.014	0.630 ± 0.014	0.683 ± 0.014	0.655 ± 0.011	0.264 ± 0.028
F-EAE	no	test	0.615 ± 0.004	0.614 ± 0.004	0.642 ± 0.003	0.634 ± 0.001	0.234 ± 0.007
GC-MC	yes	test	0.646 ± 0.001	0.645 ± 0.001	0.648 ± 0.002	0.657 ± 0.004	0.291 ± 0.002
sRGCNN	yes	test	0.633 ± 0.004	0.633 ± 0.004	0.633 ± 0.004	0.650 ± 0.005	0.266 ± 0.007
PinSage	yes	test	0.644 ± 0.003	0.643 ± 0.004	0.710 ± 0.001	0.714 ± 0.002	0.288 ± 0.006
IGMC	no	test	0.645 ± 0.007	0.639 ± 0.012	0.723 ± 0.002	0.723 ± 0.002	0.304 ± 0.004
CosMIG	no	test	0.667 ± 0.004	0.667 ± 0.004	0.742 ± 0.004	0.747 ± 0.005	0.334 ± 0.009
DGCL	no	test	0.679 ± 0.002	0.679 ± 0.002	0.737 ± 0.003	0.719 ± 0.002	0.359 ± 0.004
**BiGvCL**	no	test	**0.708 ± 0.002**	**0.707 ± 0.003**	**0.776 ± 0.002**	**0.761 ± 0.003**	**0.415 ± 0.006**

**Table 3:** Comparison of different methods on the DGIdb dataset.

**Method**	**Features**	**Split**	**Acc**	**Macro_f1**	**AUROC**	**AUPR**	**MCC**
MC	no	val	0.796 ± 0.047	0.592 ± 0.071	0.896 ± 0.030	0.620 ± 0.058	0.716 ± 0.061
GRALS	yes	val	0.862 ± 0.014	0.711 ± 0.050	0.947 ± 0.039	0.777 ± 0.074	0.812 ± 0.019
F-EAE	no	val	0.787 ± 0.024	0.313 ± 0.037	0.862 ± 0.026	0.439 ± 0.043	0.713 ± 0.033
GC-MC	yes	val	0.848 ± 0.007	0.522 ± 0.042	0.950 ± 0.007	0.575 ± 0.019	0.791 ± 0.012
sRGCNN	yes	val	0.910 ± 0.008	0.759 ± 0.068	0.939 ± 0.024	0.769 ± 0.032	0.877 ± 0.010
PinSage	yes	val	0.803 ± 0.011	0.378 ± 0.005	0.939 ± 0.009	0.486 ± 0.026	0.791 ± 0.004
IGMC	no	val	0.789 ± 0.005	0.515 ± 0.009	0.910 ± 0.001	0.502 ± 0.015	0.726 ± 0.006
CosMIG	no	val	0.819 ± 0.001	0.616 ± 0.001	0.922 ± 0.001	0.622 ± 0.006	0.762 ± 0.001
DGCL	no	val	0.930 ± 0.004	0.849 ± 0.016	0.969 ± 0.005	0.863 ± 0.002	0.906 ± 0.006
**BiGvCL**	no	val	**0.941 ± 0.002**	**0.872 ± 0.010**	**0.986 ± 0.008**	**0.894 ± 0.010**	**0.918 ± 0.002**
MC	no	test	0.796 ± 0.047	0.572 ± 0.043	0.892 ± 0.025	0.892 ± 0.025	0.701 ± 0.067
GRALS	yes	test	0.855 ± 0.009	0.689 ± 0.014	0.946 ± 0.006	0.717 ± 0.036	0.803 ± 0.011
F-EAE	no	test	0.790 ± 0.020	0.345 ± 0.024	0.345 ± 0.024	0.466 ± 0.014	0.711 ± 0.027
GC-MC	yes	test	0.832 ± 0.009	0.492 ± 0.027	0.911 ± 0.006	0.543 ± 0.025	0.769 ± 0.012
sRGCNN	yes	test	0.893 ± 0.011	0.723 ± 0.059	0.959 ± 0.016	0.757 ± 0.034	0.853 ± 0.015
PinSage	yes	test	0.836 ± 0.009	0.375 ± 0.012	0.953 ± 0.010	0.487 ± 0.034	0.773 ± 0.013
IGMC	no	test	0.791 ± 0.005	0.511 ± 0.028	0.876 ± 0.001	0.493 ± 0.018	0.493 ± 0.018
CosMIG	no	test	0.843 ± 0.001	0.638 ± 0.001	0.903 ± 0.003	0.610 ± 0.006	0.791 ± 0.001
DGCL	no	test	0.922 ± 0.002	0.826 ± 0.016	0.975 ± 0.002	0.842 ± 0.002	0.894 ± 0.003
**BiGvCL**	no	test	**0.932 ± 0.001**	**0.851 ± 0.012**	**0.985 ± 0.003**	**0.865 ± 0.004**	**0.908 ± 0.001**

Note:

MC [77]: Completes missing entries using convex optimization.

GRALS [69]: Integrates structural graph information into matrix factorization.

F-EAE [78]: Uses deep neural networks to predict interactions represented as tensors.

GC-MC [79]: Employs graph auto-encoders for message passing on bipartite interaction graphs.

sRGCNN [80]: Combines GCN and recurrent networks to capture local stationarity and reduce parameters.

PinSage [81]: Applies graph convolutions with random-walk-based neighbor sampling.

IGMC [82]: Extracts enclosing subgraphs for inductive matrix completion using GNNs.

CoSMIG [54]: Predicts relation types via communicative subgraph representation learning.

DGCL [61]: Uses dynamic hypergraph contrastive learning to extract local and global relationships

As observed in Tables 2 and 3, graph neural network-based approaches generally outperform matrix factorization-based methods such as MC, GRALS, and F-EAE, suggesting that graph structures can effectively capture the complex patterns of drug-gene interactions. On the DrugBank dataset (Table 2), BiGvCL achieves a test accuracy of 0.708, compared to 0.679 for DGCL and 0.667 for CosMIG. The AUROC reaches 0.776, while the MCC is 0.415. On the DGIdb dataset (Table 3), BiGvCL obtains a test accuracy of 0.932 and Macro_F1 of 0.851, compared to DGCL’s 0.922 and 0.826 respectively. And the standard deviations across metrics remain relatively low.

Notably, some methods not relying on additional features (BiGvCL, DGCL, CosMIG, IGMC) tend to show competitive or better performance compared to feature-based approaches. This can be attributed to their robustness in handling missing or noisy data. In real-world biomedical datasets, many entities such as antibodies, protein complexes, and certain compounds lack publicly available features. For such cases, we employ randomly initialized embeddings, allowing the model to learn representations directly from the graph structure, while feature-dependent methods struggle to effectively handle these data gaps.

### Ablation study analysis

**Figure 2:**
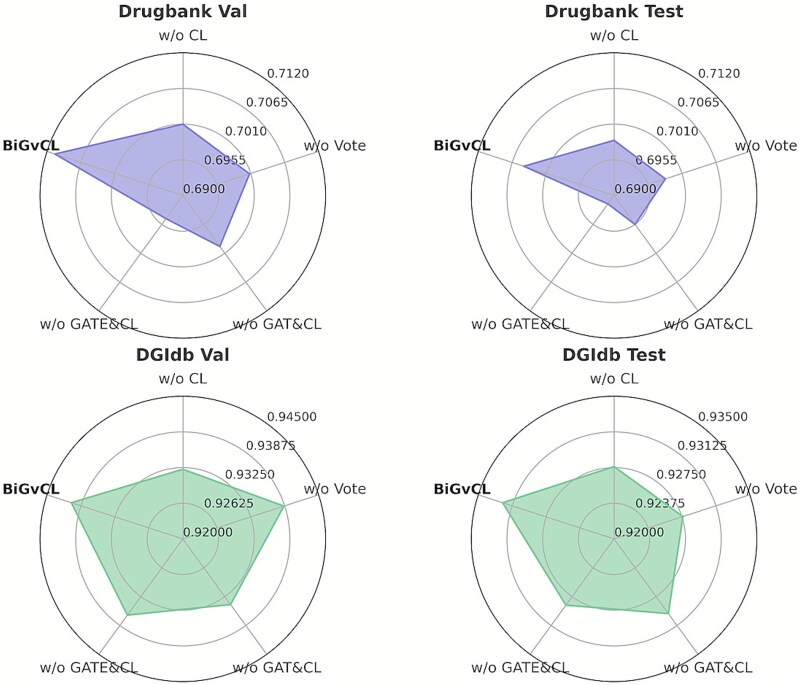
Ablation analysis of BiGvCL components.

To understand the contributions of various components in the BiGvCL model, we conducted a series of ablation experiments. Figure 2 presents performance comparisons among different model variants on both validation and test sets of the DrugBank and DGIdb datasets. Four model variants were evaluated: removal of contrastive learning (w/o CL), removal of the voting ensemble mechanism (w/o Vote), simultaneous removal of both graph attention and contrastive learning (w/o GAT&CL), and simultaneous removal of both gated graph networks and contrastive learning (w/o GATE&CL).

The full BiGvCL model consistently achieves the best performance across all settings. Quantitatively, removing contrastive learning (w/o CL) degrades test accuracy by 0.61% on DrugBank (from 0.7046 to 0.6985) and 0.47% on DGIdb (from 0.9323 to 0.9276), underscoring its role in cross-domain representation learning. Removing the voting mechanism (w/o Vote) results in 0.63% and 0.47% drops on DrugBank and DGIdb, respectively, confirming the ensemble strategy’s contribution to stability. Removing GatedGCN and contrastive learning (w/o GATE&CL) causes the largest decline on DrugBank, while removing GATLite and contrastive learning (w/o GAT&CL) leads to 0.91% degradation on DrugBank and 0.26% on DGIdb. Notably, the component importance varies across datasets: on DrugBank, GatedGCN is the most critical (1.30% contribution when combined with CL removal), while on DGIdb, contrastive learning and voting mechanism contribute equally (both 0.47%), surpassing GatedGCN’s contribution (0.37%). This difference likely stems from DGIdb’s more complex multi-relational structure (14 interaction types versus 2 in DrugBank), where contrastive learning’s ability to discriminate fine-grained semantics becomes more crucial. The radar chart in Fig. 2 visually reinforces these findings, with the purple and green areas extending furthest along the BiGvCL axis. Overall, the ablation study demonstrates that all components are indispensable.

### Hyperparameter analysis

**Figure 3:**
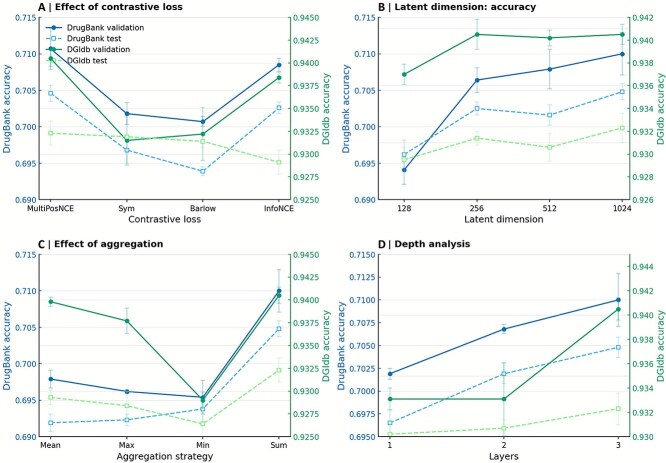
The impact of key hyperparameters of the BiGvCL model on prediction accuracy.

To investigate the impact of critical hyperparameters on the BiGvCL model, we systematically analyzed four key parameters, as illustrated in Fig. 3. For the contrastive loss function (Fig. 3A), we compared four contrastive learning losses, finding that MultiPosNCE, which treats entities sharing identical drug-gene identifiers as positive samples, achieved the best performance, outperforming Barlow by 1.54% and 0.10% on DrugBank and DGIdb test sets respectively. The embedding dimension analysis (Fig. 3B) revealed that model accuracy increased progressively with higher dimensions, with DrugBank improving from 0.6962 (128d) to 0.7048 (1024d), representing a 1.24% gain, while DGIdb performance saturated at 256 dimensions; we selected 1024 dimensions to balance performance and computational efficiency. Regarding aggregation strategies (Fig. 3C), Sum aggregation achieved the best results, outperforming Min aggregation by 1.58% and 0.63% on DrugBank and DGIdb test sets respectively, indicating that summation effectively captures complex drug-gene interaction features. The network depth analysis (Fig. 3D) showed that accuracy improved consistently with increased GNN layers, with the three-layer architecture achieving 1.19% higher test accuracy on DrugBank compared to single-layer. Based on this comprehensive analysis, we determined the optimal configuration as MultiPosNCE loss, 1024-dimensional embeddings, Sum aggregation, and 4 layers GNN architecture, achieving test accuracies of 0.7048 on DrugBank and 0.9323 on DGIdb. Given sufficient GPU resources, these parameters or higher values are recommended to optimize the model’s predictive performance for drug-gene interaction tasks while balancing computational efficiency.

### Model performance analysis on sparse networks

**Figure 4:**
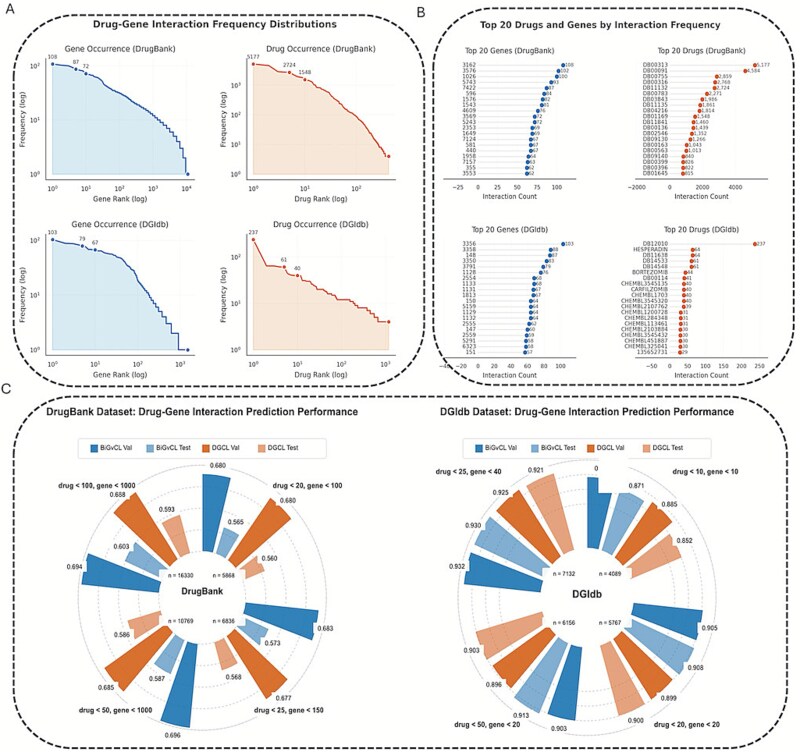
Performance analysis of the model on sparse networks. (A) Distribution of drug-gene interaction frequencies. (B) Top 20 drugs and genes by interaction count. (C) Performance comparison on DrugBank and DGIdb datasets under varying sparsity conditions.

Drug-gene interaction networks typically exhibit significant sparsity. We conducted a systematic analysis of the network sparsity characteristics. Figure 4A illustrates the distribution of drug-gene interaction frequencies in the DrugBank and DGIdb datasets. It is observed that both datasets follow a power-law distribution, characterized by a few drugs and genes involved in numerous interactions, whereas the majority of nodes maintain relatively few connections. Figure 4B further examines the top 20 most frequently interacting drugs and genes. Notably, the DGIdb dataset displays a more pronounced imbalance, with top-ranking drugs engaging in over 500 interactions, compared to ~100 interactions for the most active drugs in DrugBank.

To evaluate BiGvCL’s adaptability across networks of varying sparsity, we designed controlled experiments with constrained node numbers, as presented in Fig. 4. On the DrugBank dataset, as the network scales from a small size (drugs <20, genes <100, 5868 interactions) to a larger size (drugs <100, genes <1000, 16,330 interactions), the BiGvCL test accuracy improves from 0.565 to 0.603, consistently outperforming the DGCL benchmark. Similarly, on the DGIdb dataset, accuracy ranges from 0.871 (smallest scale: drugs <10, genes <10, 4089 interactions) to 0.930 (larger scale: drugs <25, genes <40, 7132 interactions), demonstrating larger improvements. These experimental results confirm that BiGvCL maintains improved performance across varying sparsity conditions. BiGvCL achieves accuracy of 0.908 on the DGIdb dataset even at very limited node counts, highlighting its performance in modeling real-world sparse drug-gene interaction networks.

### External testing and cross-domain knowledge validation

**Figure 5:**
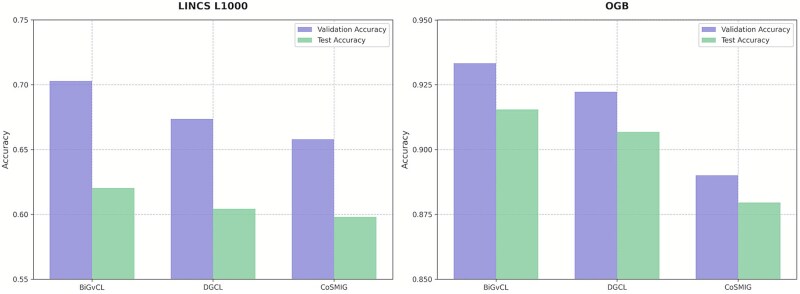
External test set and cross-domain knowledge verification.

To further validate the generalization capability of the BiGvCL model, we conducted additional evaluations using the LINCS L1000 dataset and the OGB drug-disease dataset. As depicted in Fig. 5, our proposed BiGvCL model demonstrates competitive performance across these two distinct types of datasets. On the LINCS L1000 external test dataset, BiGvCL achieves 0.6200 test accuracy, outperforming DGCL (0.6043) and CoSMIG (0.5980) by 1.57% and 2.20%, respectively. On the cross-domain OGB drug-disease dataset, BiGvCL achieves 0.9155 test accuracy, surpassing DGCL (0.9068) and CoSMIG (0.8796) by 0.87% and 3.59%. These results further confirm its generalization capabilities and cross-domain adaptability. Overall, the results from both datasets support the effectiveness of the BiGvCL method, highlighting its potential in relational learning tasks within biomedical knowledge networks.

### Integrative analysis of drug-gene interactions based on BiGvCL

**Figure 6:**
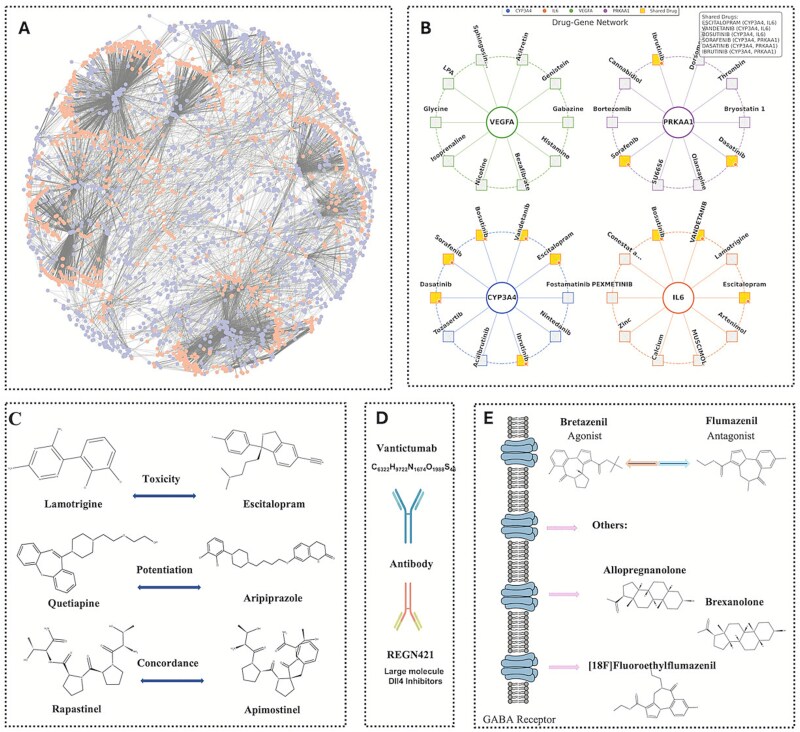
Integrated analysis of drug-gene interactions based on BiGvCL. (A) Global drug-gene interaction network. (B) Predicted drug-gene associations validated by literature. (C) Discovery of drug–drug interactions based on drug similarity. (D.) identification of antibody drugs based on drug similarity. (E) Identification of GABA receptor-related drugs based on drug similarity.

To demonstrate the practical utility of the BiGvCL model in drug repositioning and drug interaction studies, we conducted a comprehensive case study based on the model’s embedding representations, as illustrated in Fig. 6. Initially, we constructed a complete drug-gene interaction network (Fig. 6A) using training data, showing known interactions between drugs (orange nodes) and genes (blue nodes). Building upon this global network, we explored two applications.

The first application involved predicting and validating novel drug-gene interactions (Fig. 6B). Four biologically significant genes, including VEGFA, PRKAA1 CYP3A4 and IL6, were selected, and interactions predicted by the model with probabilities exceeding 0.99 were identified as high-confidence candidates. These predicted interactions did not exist in either the training or test sets, representing novel findings. Subsequent literature searches provided evidence supporting these high-confidence predictions. Specifically, we validated various predicted drugs regulating VEGFA through existing anti-angiogenesis studies (Table S1); revealed compounds influencing the cAMP signaling pathway related to PRKAA1 (Table S2). confirmed multiple potential substrates and inhibitors for the drug-metabolizing enzyme CYP3A4 (Table S3); and identified several potential modulators for the inflammatory cytokine IL6 (Table S4). These findings underscore BiGvCL’s capability not only to reproduce known interactions but also to predict previously undiscovered drug-gene relationships, verified by literature, demonstrating its potential in drug discovery applications. The second application leveraged the embedding layer of the model, identifying the top 10 most similar drug pairs to investigate drug interactions (Figs 6C–E, Table S5). In Fig. 6C, we identified three drug pairs with distinct interaction patterns: toxic interactions between Lamotrigine and Escitalopram, synergistic effects between Quetiapine and Aripiprazole, and functional consistency between Rapastinel and Apimostinel. Figure 6D highlights antibody-type drugs identified by the model, including Vanticizumab and REGN421 (DII4 inhibitors), illustrating the model’s generalization capability across diverse drug classes. Figure 6E presents various compounds associated with GABA receptors, including agonists like Bretazenil and antagonists like Flumazenil, indicating that the model captures specific receptor-associated drug patterns.

Collectively, these case studies suggest that the BiGvCL model, through its embedding representations, can predict novel drug-gene interactions, identify functional similarities among drugs, and uncover interaction patterns. Thus, BiGvCL provides a computational method for drug repositioning, combination therapy development, and target-based drug design. Relevant literature supporting these findings can be found in our supplementary materials.

### Molecular docking analysis

To further validate the practical applicability of the drug–gene interactions predicted by our model, we conducted molecular docking experiments targeting the drug-metabolizing enzyme CYP3A4. Docking analyses were performed using the online docking platform CB-Dock2 [83]. We selected Lamotrigine and Hesperadin as representative candidate compounds, which were predicted with high confidence by the BiGvCL model to interact with CYP3A4. Two protein structures (PDB IDs: 1W0E and 5VC0) were used in the docking analysis. The 1W0E structure represents CYP3A4 co-crystallized with the enzyme inhibitor metyrapone, whereas 5VC0 corresponds to CYP3A4 complexed with the antiviral drug ritonavir. These two structures capture distinct conformations of the enzyme, providing diverse binding-site characteristics.

**Figure 7:**
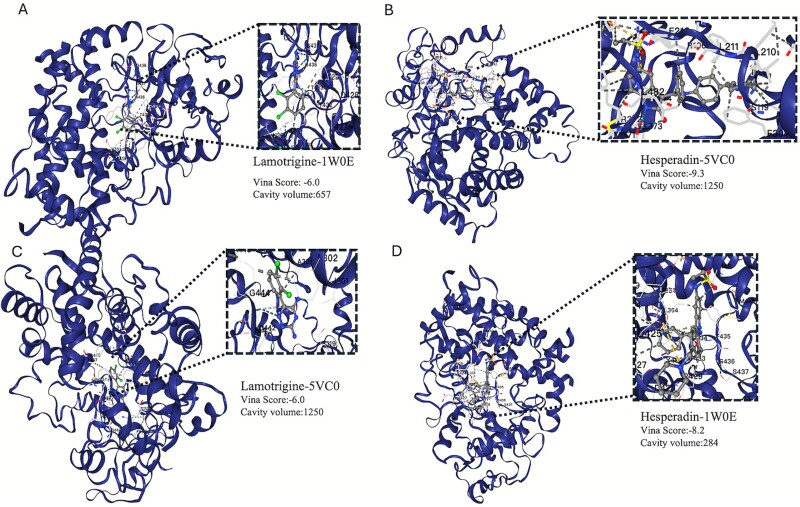
Docking poses of Lamotrigine and Hesperadin in two CYP3A4 conformations. (A) Lamotrigine bound to CYP3A4-1W0E. (B)Hesperadin bound to CYP3A4-5VC0. (C) Lamotrigine bound to CYP3A4-5VC0. (D) Hesperadin bound to CYP3A4-1W0E.

Docking results, shown in Fig. 7, indicate that Hesperadin exhibited binding affinity toward CYP3A4 (Vina scores: −9.3 with 5VC0 and −8.2 with 1W0E), suggesting its potential as a CYP3A4 ligand. Lamotrigine also demonstrated binding affinity (Vina score: −6.0 for both 5VC0 and 1W0E). These findings further highlight the potential of BiGvCL for virtual drug screening applications.

**Figure 8:**
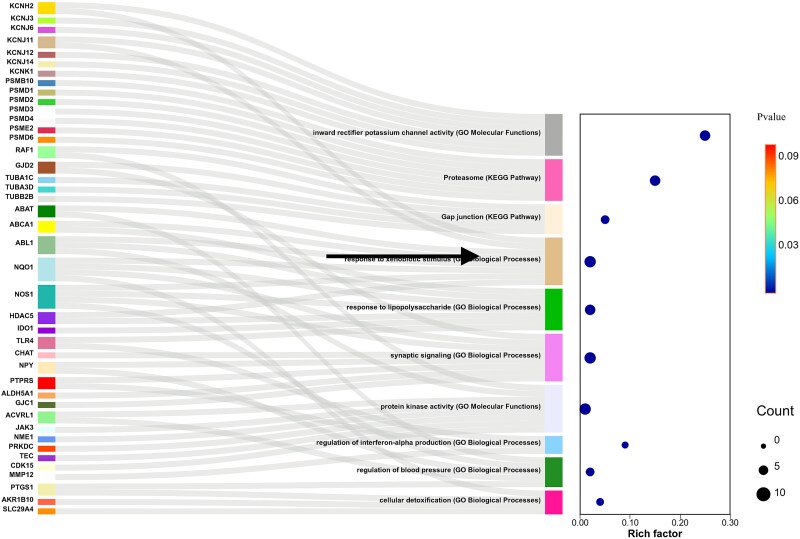
Drug-based functional annotation of predicted genes.

### Drug-based functional annotation of predicted genes

We further attempted to analyze the application potential of the BiGvCL model in enrichment analysis. In this work, we selected PAZOPANIB as a case study for validation analysis. PAZOPANIB is a potent, multi-targeted tyrosine kinase inhibitor anticancer drug that limits tumor growth by inhibiting enzymes including vascular endothelial growth factor receptor, platelet-derived growth factor receptor, c-KIT, and FGFR, with complex pharmacological mechanisms.

We performed KEGG and GO enrichment analysis on the top 100 genes highly associated with PAZOPANIB drug predicted by the BiGvCL model. Figure 8 was plotted based on Metascape results on the CNSknowall platform (https://cnsknowall.com). The enrichment analysis results showed that the predicted genes mainly participate in several key functional modules: the most significantly enriched pathway was ‘inward rectifier potassium channel activity’ (GO Molecular Functions, Rich factor = 0.25, P < 1 × 10^−13^), involving seven key genes; followed by the proteasome pathway (KEGG Pathway) and gap junction pathway; additionally including immune inflammatory response, protein kinase activity, and synaptic signaling functional modules.

## Conclusions

In this study, we introduced BiGvCL, a novel bipartite graph-based cross-domain contrastive learning framework designed for predicting DGIs. BiGvCL integrates a GATLite, a GatedGCN, and a contrastive learning strategy to capture complex interaction patterns. Evaluations on benchmark datasets (DrugBank, DGIdb, LINCS L1000, and OGB) highlighted BiGvCL’s stability and cross-domain generalization capability. Ablation studies further validated the contributions of the contrastive learning and gating mechanisms, while systematic hyperparameter analyses determined optimal configurations balancing predictive accuracy with computational efficiency. Additionally, BiGvCL demonstrated adaptability under sparse network conditions and predicted novel drug-gene interactions with supporting evidence from existing literature. Despite these strengths, our approach has inherent limitations, such as reliance solely on network topology without explicit molecular annotations, and the constraints imposed by its transductive learning strategy, limiting predictions for unseen entities. In the future, we will extend BiGvCL to inductive learning scenarios and integrate multimodal biomedical data to enhance its predictive performance and generalization capability.

**Key Points**

- Developed a novel graph contrastive learning model to capture drug–gene relationships based exclusively on topology.
- Achieved competitive metrics across DrugBank, DGIdb, LINCS L1000, and OGB biokg benchmarks.
- Demonstrated consistent robustness in predicting interactions within sparse and small-scale networks.
- Provided molecular docking validation supporting the biological plausibility of predicted interactions.
- Enabled identification of potential drug repositioning targets without requiring external biochemical or genomic data.

## Supplementary Material

bbaf710_Supplemental_Files

## Data Availability

The data and code in this study are openly accessible on GitHub (https://github.com/heshida01/BiGvCL).
