# Supplementary material for: BiGvCL: bipartite graph-based cross-domain contrastive learning model for the predicting drug-gene interactions
Source: Brief Bioinform. 2026 Jan 28;27(1):bbaf710. doi: 10.1093/bib/bbaf710 (PMC12848949; doi:10.1093/bib/bbaf710)
Supplement: bbaf710_Supplemental_Files [file bbaf710_supplemental_files.pdf]

Supplementary Information

Table 1. BiGvCL-Predicted Candidate Drugs Targeting VEGFA

Drug	Gene	Reference
Gabazine	VEGFA	https://doi.org/10.1111/jnc.16182
Genistein	VEGFA	https://doi.org/10.1155/2024/5338212
Acitretin	VEGFA	https://doi.org/10.5352/JLS.2009.19.3.327
Oxymetazoline	VEGFA	https://doi.org/10.14670/HH-18-399
Sphingosine 1-Phosphate	VEGFA	https://doi.org/10.1016/j.celrep.2019.11.036
Alitretinoin	VEGFA	https://doi.org/10.3390/ijms24119654
LPA	VEGFA	https://doi.org/10.1016/j.cellsig.2008.02.009
Glycine	VEGFA	https://doi.org/10.1038/s41598-017-15246-3
Isoprenaline	VEGFA	https://doi.org/10.1186/s12885-017-3894-0
Histamine	VEGFA	https://doi.org/10.4049/jimmunol.177.10.7322

Note:

Ten drugs are predicted by BiGvCL with high confidence to modulate the angiogenic factor VEGFA, and each interaction is corroborated by peer-reviewed studies. This highlights the framework's potential to identify biologically meaningful relationships using DGI network topology.

Table 2. BiGvCL-Predicted Candidate Drugs Targeting PRKAA1

Drug	Gene	Reference
Bryostatin1	PRKAA1	https://doi.org/10.1371/journal.pone.0011160
Thrombin	PRKAA1	https://doi.org/10.1128/MCB.00383-06
Dorsomorphin	PRKAA1	https://doi.org/10.1080/15548627.2015.1034406
Ibrutinib	PRKAA1	https://doi.org/10.1161/JAHA.123.032357
Cannabidiol	PRKAA1	https://doi.org/10.1002/mnfr.202300446
Bortezomib	PRKAA1	https://doi.org/10.1007/s00280-014-2451-7
Sorafenib	PRKAA1	https://doi.org/10.1016/j.chembiol.2017.05.021
SU6656	PRKAA1	https://doi.org/10.1016/j.chembiol.2017.05.021
Olanzapine	PRKAA1	https://doi.org/10.1007/s10571-011-9663-8
Dasatinib	PRKAA1	https://doi.org/10.18632/oncotarget.1628

Note:

For the gene PRKAA1, BiGvCL predicts 10 high-confidence drug interactions (posterior probability > 99%), all of which are corroborated by peer-reviewed studies, offering valuable guidance for metabolic-disease drug development.

Table 3. BiGvCL-Predicted Candidate Drugs Targeting CYP3A4

Drug	Gene	Reference
Fostamatinib	CYP3A4	https://go.drugbank.com/drugs/DB12010
Escitalopram	CYP3A4	https://go.drugbank.com/drugs/DB01175
Vandetanib	CYP3A4	https://doi.org/10.2165/11586980-000000000-00000
Bosutinib	CYP3A4	https://doi.org/10.1007/s00228-016-2108-z
Sorafenib	CYP3A4	https://go.drugbank.com/drugs/DB00398
Dasatinib	CYP3A4	https://doi.org/10.1124/dmd.108.025932
Tozasertib	CYP3A4	https://doi.org/10.3390/ijms14022707
Ibrutinib	CYP3A4	https://go.drugbank.com/drugs/DB09053
Nintedanib	CYP3A4	https://go.drugbank.com/drugs/DB09079
Brigatinib	CYP3A4	https://go.drugbank.com/drugs/DB12267

Note:

Ten compounds—including clinically important kinase inhibitors—are flagged by BiGvCL as CYP3A4 interactors with probability > 0.99, confirming the topology-only model's precision.

Table 4. BiGvCL-Predicted Candidate Drugs Targeting IL6

Drug	Gene	Reference
Escitalopram	IL6	https://doi.org/10.1016/j.pnpbp.2023.110762
Lamotrigine	IL6	https://doi.org/10.1016/j.jneuroim.2018.06.008
Vandetanib	IL6	https://doi.org/10.1021/acsomega.2c02794
Bosutinib	IL6	https://doi.org/10.1042/BJ20141165
Conestat alfa	IL6	https://doi.org/10.3389/fimmu.2020.02072
Pexnetinib	IL6	https://doi.org/10.1182/blood.V110.11.4798.4798
Calcium	IL6	https://doi.org/10.1371/journal.pone.0092649
Muscimol	IL6	https://doi.org/10.1136/svn-2016-000012
Artenimol	IL6	https://doi.org/10.3892/ijmm.2010.580
Zinc	IL6	https://doi.org/10.1017/S0007114521000192

Note:

BiGvCL identifies ten small molecules that likely influence pro-inflammatory cytokine IL-6 with probability > 0.99; literature verification for each DGI demonstrates the framework's value in anti-inflammatory drug repurposing.

Table 5. Top 10 High-Similarity Drug–Drug Pairs

Drug	Drug	Reference
Vantictumab	REGN421	https://doi.org/10.3389/fonc.2016.00115
Lamotrigine	Escitalopram	https://go.drugbank.com/drugs/DB00555
Bretazenil	Flumazenil	https://en.wikipedia.org/wiki/Bretazenil
Allopregnanolone	Flumazenil	https://doi.org/10.1016/0014-2999(95)00311-8
Allopregnanolone	[3H]CGS8216	https://doi.org/10.1016/j.neuroscience.2010.07.037; https://doi.org/10.1016/0024-3205(82)90403-9
Allopregnanolone	[18F]Fluoroethylflumazenil	https://doi.org/10.1016/j.nucmedbio.2003.09.003
Rapastinel	Apimostinel	https://doi.org/10.3390/ph18020157
Allopregnanolone	RO15-4513	https://doi.org/10.1016/j.neuroimage.2014.05.035
Enprofylline	Escitalopram	N/A
Quetiapine	Aripiprazole	https://go.drugbank.com/drugs/DB01224

Note:

This table lists the ten drug pairs with the highest similarity scores. Nine of these pairs are supported by literature evidence (see the "Reference" column), demonstrating the potential of the model for assessing pharmacological redundancy, common mechanisms, or potential interaction risks, thus facilitating subsequent drug development and clinical research.
